# Risk correlation analysis of serum adiponectin (APN), D-dimer (DD) and the neutrophil - lymphocyte ratio (NLR) with the prognosis of diabetic foot ulcer infection

**DOI:** 10.5937/jomb0-60169

**Published:** 2026-03-17

**Authors:** Guodong Guo, Xiaohui Zhang, Peng Fang, Kangkanng Li, Chaoyang Wang, Gang Liu, Huijuan Ma

**Affiliations:** 1 Department of Orthopaedics, Nanjing Jinling Hospital, Affiliated Hospital of Medical School, Nanjing University, China; 2 Department of Anesthesiology, Nanjing Jinling Hospital, Affiliated Hospital of Medical School, Nanjing University, China; 3 Endocrinology Department, Shanghai Jiao Tong University Affiliated Renji Hospital, China; 4 Department of Neurology, Sir Run Run Hospital, Nanjing Medical University, China

**Keywords:** diabetic foot ulcer, adiponectin (APN), D-dimer (D-D), neutrophil-lymphocyte ratio (NLR), dijabetičko stopalo, adiponektin (APN), D-dimer (D-D), odnos neutrofila i limfocita (NLR)

## Abstract

**Background:**

To investigate the connections between diabetic foot ulcer infection patient prognosis and disease severity, we measured serum adiponectin (APN) and D-dimer (D-D) levels and the neutrophil lymphocyte ratio (NLR).

**Methods:**

A total of 292 patients with diabetic foot ulcer infection admitted to our hospital from February 2024 to February 2025 were selected and divided into mild (n = 95), moderate (n = 139) and severe (n = 58) patients according to the severity of the disease. The levels of serum APN and D-D and the NLR in patients with different severity levels were compared. To analyse the serum APN, D-D, and NLR, patients were followed up for one year, and the prognoses of the patients were statistically analysed.

**Results:**

Comparisons of the serum APN, D-D and NLR levels among patients with different disease severities revealed that the serum APN levels in patients with moderate disease were lower than those in patients with mild disease (P&lt;0.05). The serum APN of severe patients was 5.35± 0.98, and that of moderate patients was 7.64±1.25, which was lower than that of mild patients (9.19±1.73; P&lt;0.05), and the serum APN level of severe patients was lower than that of moderate patients (P&lt;0.05). Comparisons of the serum D-D and NLR levels revealed that the serum D-D and NLR in severe patients were 3.49±0.72 and 2.86±0.58, respectively, and those in moderate patients were 3.02±0.63 and 2.24±0.46, respectively, both of which were greater than those in mild patients, which were 2.43±0.51 and 1.71±0.33 (P&lt;0.05). The levels of serum D-D and the NLR were greater in severe patients than in moderate patients (P&lt;0.05). Spearman correlation analysis revealed that the severity of disease in diabetic foot ulcer infection patients was positively related to the serum D-D level and NLR (r=0.387, P&lt;0.001; r=0.461, P&lt;0.001) and negatively related to the serum APN level (r=-0.414, P&lt;0.001). ROC analysis revealed that the optimal cutoff values of the serum APN, D-D and NLR for predicting poor prognosis in patients were 5.73 mg/L, 3.06 mg/L and 2.12, respectively; the sensitivities were 78.57%, 82.14% and 85.71%, respectively; and the specificities were 76.56%, 67.19% and 73.44%, respectively. The areas under the curve (AUCs) were 0.793, 0.784, and 0.818, respectively. The specificity and AUC of the combination of the three methods were 98.44% and 0.918, respectively.

**Conclusions:**

Serum APN and D-D levels and the NLR in patients with diabetic foot ulcer infection are related to disease severity. The serum APN, D-D and NLR can be used as sensitive indicators for predicting poor patient prognosis.

## Abstract (sr)

**Uvod:**

Cilj ovog istraživanja bio je da se ispita povezanost između prognoze kod pacijenata sa infekcijom dijabetičkog stopala i težine bolesti, putem merenja nivoa serumskog adiponektina (APN) i D-dimera (D-D), kao i odnosa neutrofila i limfocita (NLR).

**Metode:**

U studiju je uključeno 292 pacijenta sa infekcijom dijabetičkog stopala, primljena u nasu bolnicu od februara do februara 2025. godine. Pacjenti su, prema težini bolesti, podeljeni u tri grupe: blaga (n = 95), umerena (n = 139) i teska (n = 58). Upoređivani su nivoi serumskog APN i D-D, kao i NLR, među pacijentima različitih težina bolesti. Radi analize ovih parametara, pacijenti su praćeni tokom jedne godine, a njihova prognoza je statistički obrađena.

**Rezultati:**

Poređenjem nivoa serumskog APN, D-D i NLR među grupama, utvrđeno je da su vrednosti APN kod pacijenata sa umerenom bolesću bile niže nego kod blagih pacijenata (P&lt;0,05). Kod teskih pacijenata nivo APN iznosio j'e 5,35±0,98, a kod umerenih 7,64±1,25, sto j'e takođe bilo niže u poređenju sa blagim pacijentima (9,19±1,73; P&lt;0,05). Nivo a Pn kod teskih pacij'enata bio je niži nego kod umerenih (P&lt;0,05). Analiza nivoa D-D i NLR pokazala je da su kod teskih pacijenata vrednosti bile 3,49±0,72 i 2,86±0,58, dok su kod umerenih iznosile 3,02±0,63 i 2,24±0,46 - obe vrednosti vise nego kod blagih pacjenata (2,43±0,51 i 1,71 ±0,33; P&lt;0,05). Nivoi D-D i NLR bili su značajno visi kod teskih nego kod umerenih pacijenata (P&lt;0,05). Spearmanova korelaciona analiza pokazala je da je težina bolesti pozitivno povezana sa nivoom D-D (r=0,387, P&lt;0,001) i NLR (r=0,461, P&lt;0,001), a negativno povezana sa nivoom APN (r=-0,414, P&lt;0,001). ROC analiza je pokazala da su optimalne granične vrednosti APN, D-D i NLR za predviđanje lose prognoze bile 5,73 mg/L, 3,06 mg/L i 2,12, sa osetlji-vosću od 78,57%, 82,14% i 85,71% i specifičnošću od 76,56%, 67,19% i 73,44%. Povrsine ispod krive (AUC) iznosile su 0,793, 0,784 i 0,818. Kombinacijom sva tri parametra dobijena je specifičnost od 98,44% i AUC od 0,918.

**Zaključak:**

Nivoi serumskog APN i D-D, kao i NLR, kod pacijenata sa infekcijom dijabetičkog stopala povezani su sa težinom bolesti. Ovi parametri mogu poslužiti kao osetljivi pokazatelji za predviđanje nepovoljne prognoze kod pacijenata.

## Introduction

The long-term hyperglycemic state and insulin resistance of diabetic patients can promote the release of inflammatory factors in the body, leading to peripheral neuropathy or lower extremity vascular disease, and easily causing diabetic foot ulcers (DFUs) [1][2][3]. Most patients with DFU are accompanied by infections of varying degrees, resulting in a series of problems, such as lower extremity pain, soft tissue destruction and joint deformity [4][5][6]. In severe cases, it can cause systemic infections, seriously affecting the physical health of patients. Clinical studies have shown that early assessment of the condition of patients with DFU infection and effective treatment can improve patient prognosis. Adiponectin (APN) is a protective adipokine that can increase insulin sensitivity and may be involved in the occurrence and development of DFU infection [7]. D-dimer (D-D) is a product of fibrin degradation and can reflect the hypercoagulable state of the body [8]. The neutrophil-to-lymphocyte ratio (NLR) is an inflammatory indicator that can stably reflect the inflammatory status of the body and is related to the prognosis of inflammatory diseases such as systemic lupus erythematosus and diabetes [9]. Adiponectin, an adipokine with anti-inflammatory and insulin-sensitising properties, is implicated in impaired wound healing in individuals with diabetes. Hypoadiponectinemia is correlated with microvascular dysfunction and infection risk in DFU patients [10][11][12].

In this study, 292 patients with DFU infection admitted to our hospital were selected to explore the changes in serum APN, D-D and NLR levels and their relationships with the severity of the disease and patient prognosis.

## Materials and methods

### Clinical data

A total of 292 patients with DFU infection admitted to our hospital from February 2024 to February 2025 were selected, including 158 males and 134 females, with an age of 55.73±9.34 years, ranging from 24-78 years.

Inclusion criteria: (1) Met the diagnostic criteria for DFU infection; (2) Had a fasting blood glucose level >7 mmol/L; (3) Had a duration of diabetes of 1-15 years.

Exclusion criteria: (1) Other vascular surgical diseases; (2) Severe dysfunction of the heart, liver or kidneys; (3) Mental illness; (4) Pregnancy or lactation; (5) Rheumatic immune diseases.

### Data collection

Data such as sex, age, duration of diabetes, smoking history, history of lower extremity interventional surgery, ulcer area, combined hypertension, and severity of the disease were collected and statistically analysed. All patients were given standard treatments, such as hypoglycemic, antibacterial and blood circulation improvements. The patients were followed up for one year to statistically analyse the prognosis.

### Detection methods for serum indicators

The levels of serum APN and D-D were determined via enzyme-linked immunosorbent assay. Neutrophils and lymphocytes were determined with a Beckman DxH800 fully automatic haematology analyser in the United States, and the NLR was calculated. Simultaneously, fasting blood glucose, glycated haemoglobin, C-reactive protein, fibrinogen, white blood cell count, total cholesterol, triglycerides and albumin were detected. Serum samples were centrifuged at 3,000 rpm for 15 min, aliquoted, and stored at -80°C until analysis. All assays included internal controls and were performed in duplicate.

### Observation indices

(1) The levels of serum APN, D-D and the NLR in patients with different degrees of severity were compared. (2) Serum APN, D-D, and NLR levels in DFU-infected patients were correlated with disease severity via Spearman correlation analysis. (3) Univariate analysis was used to analyse the factors influencing the levels of serum APN, D-D and the NLR in patients with DFU infection. (4) The risk factors impacting the poor outcome of individuals with DFU infection were examined via logistic regression analysis. (5) Analysis of the value of the serum APN, D-D and the NLR in predicting the poor prognosis of patients was performed.

### Criteria for determining the severity of illness

(1) Mild: redness, swelling, suppuration, pain, and two or more hard lumps, with redness and swelling at the ulcer edge less than 2 cm; (2) Moderate: the overall condition is stable, with redness and swelling at the ulcer margin >2 cm and subfascial infection spreading; (3) Severe: a systemic inflammatory response occurs.

Good prognosis: clinical symptoms disappear, and there is no recurrence within one year; poor prognosis: nonhealing of the wound, recurrence within one year, amputation, etc.

### Laboratory measurements

Venous blood samples were collected from all patients after an overnight fast. Serum adiponectin (APN) was quantified via enzyme-linked immunosorbent assay (ELISA) (Human Adiponectin ELISA Kit, R&D Systems, Cat# DRP300). The absorbance was measured at 450 nm on a BioTek Synergy H1 microplate reader. The intra- and interassay coefficients of variation (CVs) were <5% and <8%, respectively.

D-dimer (DD): D-dimer was analysed via an immunoturbidimetric assay (Siemens Healthineers, Atellica COAG 360 System). The reference range was 0-0.5 mg/L FEU.

Neutrophil Lymphocyte Ratio (NLR): This ratio was calculated from complete blood counts (CBCs) performed on a Sysmex XN-9000 haematology analyser within 2 hours of sample collection.

### Statistical analysis

SPSS 22.0 software was used to examine the data. Analysis of variance was performed in one direction for comparisons between groups. A 2 test was conducted, and count statistics are shown as (Cases (%)). To determine the value of serum APN, D-D, and the NLR in predicting poor patient prognosis, Spearman analysis was used to examine correlations. P values less than 0.05 were regarded as statistically significant.

## Results

Comparison of serum APN, D-D, and NLR levels in patients with DFU of different severity levels. Comparisons of the serum APN, D-D, and NLR levels among patients with DFU of different severities revealed statistically significant differences. Additionally, patients with severe symptoms had lower blood APN levels than those with moderate symptoms did, and patients with severe symptoms had lower serum APN levels than those with mild symptoms did. According to the NLR and serum D-D levels, patients with severe and moderate conditions had higher levels than those with mild symptoms, and patients with severe conditions had higher levels than those with moderate illnesses. Spearman correlation analysis revealed that the severity of disease in DFU-infected patients was positively correlated with the serum D-D level and the NLR (r=0.387, P<0.001; r=0.461, P<0.001) and inversely correlated with the serum ApN level (r=-0.414, P<0.001) (Table 1).

**Table 1:** Comparison of serum APN, D-D and NLR levels in patients with diabetic foot of different severity (x̄±s).

Group	Number of cases	APN (mg/L)	D-D (mg/L)	NLR
Mild	95	9.19±1.73	2.43±0.51	1.71±0.33
Moderate	139	7.64±1.25	3.02±0.63	2.24±0.46
Severe	58	5.35±0.98	3.49±0.72	2.86±0.58
F value	-	43.418	17.963	37.386
P value	-	<0.001	<0.001	<0.001

### Univariate analysis of the factors influencing the levels of serum APN and D-D and the NLR in patients with DFU infection

Univariate analysis revealed that the composition ratios of severe conditions and the serum fasting blood glucose, glycated haemoglobin, fibrinogen, D-D, and NLR levels were greater than those of patients with good prognoses, and patients with poor prognoses had lower APN levels than did those with excellent prognoses (Table 2).

**Table 2:** Univariate Analysis of Factors influencing Serum APN, D-D and NLR Levels in Patients with diabetic foot ulcer Infection (x̄±s).

Factor	Poor prognosis (n=88)	Good prognosis (n=204)	Statistical value	P value
Gender			0.127	0.722
Male	50 (57.14)	108 (53.13)		
Female	38 (42.86)	96 (46.87)		
Age (Years)	56.38±8.17	55.24±8.56	0.596	0.553
Course of diabetes (Years)	8.79±1.54	8.36±1.33	1.359	0.178
History of smoking				
Yes	46 (53.57)	98 (48.44)	0.205	0.650
No	42 (46.43)	106 (51.56)		
History of lower extremity <br>interventional surgery				
Yes	22 (25.00)	34 (17.19)	0.755	0.385
No	66(75.00)	170 (82.81)		
Ulcer area (cm^2^)				
<10	19 (67.86)	52 (81.25)	1.983	0.159
≥10	9 (32.14)	12 (18.75)		
Combined with hypertension				
Yes	24 (28.57)	43(21.88)	0.480	0.488
No	64 (71.43)	161 (78.12)		
Severity of the illness				
Severe	53 (60.71)	41 (20.31)	14.709	0.001
Moderate	22 (25.00)	116 (57.81)		
Mild	13 (14.29)	47 (21.88)		
Fasting blood glucose (mmol/L)	10.13±1.79	8.74±1.62	3.667	<0.001
Glycated haemoglobin (%)	9.36±1.62	7.98±1.46	4.034	<0.001
C-reactive protein (mg/L)	44.57±8.64	41.81±7.85	1.505	0.136
Fibrinogen (g/L)	4.18±0.73	3.26±0.64	6.076	<0.001
White blood cell count (x10^9^/L)	10.02±1.78	9.73±1.67	0.7516	0.454
Total cholesterol (mmol/L)	4.61±0.88	4.84±0.93	1.109	0.270
Triglyceride (mmol/L)	1.54±0.31	1.64±0.35	1.304	0.196
Albumin (g/L)	32.46±5.73	34.08±6.11	1.192	0.236
APN (mg/L)	5.18±0.94	8.22±1.47	10.063	<0.001
D-D (mg/L)	3.36±0.63	2.84±0.56	3.944	<0.001
NLR	2.57±0.52	1.89±0.38	7.031	<0.001

### Logistic regression analysis was used to analyse the risk factors influencing the poor prognosis of patients with DFU infection

The levels of severe illness, serum fasting blood glucose, glycated haemoglobin, fibrinogen, APN, D-D, and the NLR were taken as independent variables and assigned values. Logistic multivariate regression analysis was conducted with poor prognosis (no = 0, yes = 1) as the dependent variable. The results revealed that severe illness and serum levels of glycated haemoglobin, APN, D-D, and NLR were all independent risk factors affecting the poor prognosis of patients with diabetic DFU infection (Table 3).

**Table 3:** Logistic multivariate analysis of independent risk factors influencing poor prognosis in patients with diabetic foot ulcer infection.

Factor	β	SE	Waldx^2^	P value	OR	95%CI
APN	1.431	0.389	13.533	<0.001	4.183	1.503~11.645
D-D	1.257	0.446	7.943	<0.001	3.515	1.263~9.785
NLR	1.306	0.364	12.873	<0.001	3.691	1.326~10.277
Severe illness	1.183	0.517	5.236	0.004	3.264	1.234~8.633
Glycated hemoglobin	1.204	0.483	6.214	<0.001	3.333	1.051~10.574

### Value of serum APN, D-D and NLR levels in predicting poor patient prognosis

ROC analysis revealed that the optimal cutoff points of the serum APN, D-D and NLR for predicting poor prognosis in patients were 5.73 mg/L, 3.06 mg/L and 2.12, respectively. The specificity and AUC of the combination of the three methods were 98.44% and 0.918, respectively (Table 4).

**Table 4:** The role of serum APN, D-D, and NLR levels in predicting a bad outcome for diabetic foot ulcer patients.

Indicator	Optimal cutoff point	Sensitivity (%)	Specificity (%)	AUC	95%CI
APN (mg/L)	5.73	78.57	76.56	0.793	0.696~0.870
D-D (mg/L)	3.06	82.14	67.19	0.784	0.686~0.863
NLR	2.12	85.71	73.44	0.818	0.723~0.890
The combination<br>of the three	-	78.57	98.44	0.918	0.842~0.965

## Discussion

The long-term hyperglycemic state of diabetic patients leads to changes in various physical and chemical properties of the blood, which may be a chronic inflammatory process. Inflammatory factors have an important influence on insulin resistance in the body [13]. Moreover, chronic inflammation changes hemodynamics by damaging vascular endothelial cells. The dual action of inflammatory factors and hyperinsulinemia can cause ischemia of lower extremity tissues and stenosis and occlusion of vascular lumens [14][15][16]. DFU results in peripheral vasculature and neuropathy of the lower extremities and eventually induces DFU infection [17]. An early understanding of the changes in the condition of patients with DFU infection and prevention of disease progression can improve patient prognosis.

Patients with severe and moderate conditions in this study had lower serum APN levels than those with mild conditions did. Patients with severe conditions had lower serum APN levels than those with moderate conditions did. Additionally, patients with severe and moderate conditions had higher serum D-D and NLR levels than patients with mild conditions did, and the serum D-D and NLR levels of patients with severe conditions were greater than those of patients with moderate conditions. Moreover, the severity of the disease in patients with DFU infection was negatively correlated with the serum APN level [18][19][20]. It can promote the phosphorylation of Akt kinase in the postinsulin signalling pathway, thereby increasing insulin activity and regulating the inflammatory response of vascular endothelial cells. Studies have shown that low levels of APN can induce inflammatory cascade reactions, induce monocyte expression of chemokines, promote the aggregation of inflammatory cells at the lesion site, and facilitate the occurrence and development of peripheral neuropathy [21]. D-D is a metabolic degradation product of cross-linked fibres that can cause direct damage to the vascular walls of diabetic patients and stimulate the proliferation of smooth muscle cells, leading to vascular endothelial dysfunction [22]. In patients with DFU infection, many mature neutrophils in the bone marrow are activated and release large amounts of inflammatory factors, damaging the vascular endothelium. Moreover, lymphocytes are affected by inflammatory factors and accelerate apoptosis [23]. In addition, the hyperglycemic state of patients increases reactive oxygen species, accelerating the oxidative damage of DNA in lymphocytes and thereby increasing the NLR. In this study, moderate to severe illness and serum glycated haemoglobin, APN, D-D, and NLR levels were found to be independent risk factors affecting the poor prognosis of patients with DFU infection. ROC analysis revealed that the optimal cutoff points of the serum APN, D-D, and NLR for predicting poor patient prognosis were 5.73 mg/L, 3.06 mg/L, and 2.12, respectively.

The specificity and AUC of the combination of the three methods were 98.44% and 0.918, respectively. The NLR can reflect the dynamic balance of neutrophils and lymphocytes. Compared with a single inflammatory indicator, the NLR is less affected by external stimulating factors and can more stably reflect the inflammatory status of the body [24]. Research reports [25][26][27] indicate that D-D in patients with DFU infection is closely related to the degree of infection, which is consistent with the results of this study. Consistent with the findings of this investigation, another study [28] reported a correlation between the degree of infection and the serum APN level in patients with DFU infection. The clinical detection of serum APN, D-D and NLR levels is convenient and fast, and the combined prediction of these three indicators can improve the predictive value. Our finding of decreasing APN with worsening DFU severity aligns with studies linking hypoadiponectinemia to impaired angiogenesis and persistent inflammation in chronic wounds [29][30][31].

This study included only 92 patients with DFU infection. The sample size was small, which could have significantly affected the statistical results. The sample size still needs to be expanded to confirm the research results.

The levels of serum APN, D-D and NLR in patients with DFU infection are related to the severity of the disease. The serum APN, D-D and NLR can be used as sensitive indicators for predicting poor patient prognosis.

## Dodatak

### Authors' contributions

Guodong Guo and Xiaohui Zhang contributed equally to this work.

### Fund

2023 Life and Health Technology Special Project of Nanjing [grant number 202305018].

### Conflict of interest statement

All the authors declare that they have no conflict of interest in this work.
